# The BJGP Open Top 10 Most Read Research Articles of 2024: an editorial

**DOI:** 10.3399/BJGPO.2025.0047

**Published:** 2025-03-28

**Authors:** Alice M Harper, Hajira Dambha-Miller

**Affiliations:** 1 BJGP Open, Royal College of GPs, London, UK

**Keywords:** editorial, primary healthcare, general practitioners

The year 2024 has marked another successful chapter for *BJGP Open*, characterised by a continued expansion in both the volume and diversity of manuscripts submitted. Our journal has attracted contributions from an increasing number of countries, covering a broad spectrum of topics that are highly relevant to international primary care. From examining parental perspectives on childhood asthma care to exploring Dutch GPs’ approaches to addressing obesity, we have been keen to understand the areas of greatest interest to our readership. This editorial reflects on the articles that have emerged as the *Top Ten Most Read Research Articles of 2024*, offering a valuable insight into the issues that are shaping the field of primary care worldwide.

International primary care continues to face persistent challenges that affect both patients and healthcare professionals. Over the past year, research has provided new perspectives on the factors influencing care delivery in general practice. For instance, Pereira *et al* explored the impact of nursing practice environments on patient safety and found limited evidence on their direct influence. However, they highlighted that leadership, communication, and organisational culture and policies may play a critical role in shaping these outcomes.^1^ Expanding on the theme of leadership, Bhatti *et al* identified that transparent communication, opportunities for collaborative decision-making, and staff recognition improved employee satisfaction, motivation, and care delivery.^2^ Effective teamworking and building ongoing therapeutic relationships with patients are central to successful general practice. Fox *et al* identified seventeen distinct interventions that have been trialled to enhance relational continuity in UK general practice.^3^ Relational continuity provides an invaluable opportunity to understand patients holistically and consider the psychosocial context of their health concerns. A systematic review of patient perspectives revealed that integrating social determinants of health into primary care electronic health records was widely accepted by patients, thus providing a means of improving care outcomes through more comprehensive understanding.^4^

In our 2022 and 2023 editorials, remote consulting and telehealth emerged as prominent areas of research. Much of this work focused on synchronous telemedicine, such as real-time video and phone consultations, rather than asynchronous telemedicine, which occurs through secure messaging systems that are subsequently reviewed. Leighton *et al* investigated the uses and effectiveness of asynchronous telemedicine, evaluating its quality in relation to healthcare domains. They concluded that while asynchronous telemedicine provides quality care, it is limited by increased workload and inefficient workflow compared to face-to-face consultations.^5^ One promising application of asynchronous telemedicine could be in primary care testing, such as for Prostate-Specific Antigen (PSA) testing. A recent evaluation of the Prostate Cancer UK online risk checker showed that the tool helped men at risk make informed decisions about PSA testing.^6^

Continuing the technology theme, artificial intelligence (AI) has gained significant attention among our readers this year. Clough *et al* demonstrated that AI-written discharge summaries were of equivalent quality to those drafted by resident doctors who had completed the UK Foundation Programme.^7^ This finding holds particular relevance for the growing number of resident doctors applying for UK-based GP training.^8^ The selection process for this training has evolved from using a selection centre to the current Multi-Specialty Recruitment Assessment (MSRA). Tiffin *et al* reported that all components of this selection process predict future performance in the Clinical Skills Assessment (CSA), now the Simulated Consultation Assessment (SCA), a crucial part of the Membership of the Royal College of General Practitioners examination.^9^

AI may also have the potential to support primary care prescribing in the future. Homan *et al* found that there is room for improvement in the prescribing of direct-acting oral anticoagulants (DOACs), with essential patient information such as creatinine clearance often not being recorded for a substantial number of patients in England.^10^ Prescribing decisions are influenced not only by patient parameters but also by relevant clinical guidelines and expert consensus, particularly in the context of antimicrobial stewardship. A nationwide retrospective cohort study in Norway of antibiotic prescriptions for men diagnosed with acute cystitis in primary care supported the use of narrow-spectrum antibiotics as a first-line treatment.^11^

Our *Top 10 Most Read Research Articles of 2024* underscore the ongoing relevance of understanding the environments in which we work, the role of technology and online tools in clinical practice, the selection of the next generation of GPs, and the need for safe, evidence-based prescribing practices. As we look to the future, we eagerly anticipate further submissions addressing critical questions in international primary care, with the aim of advancing the quality of care provided to patients around the globe.

## Data

N/A

## References

[1] de Abreu Pereira SC, Lopes Ribeiro OMP, Fassarella CS, Ferreira Santos EJ (2024). The impact of nursing practice environments on patient safety culture in primary health care: a scoping review. BJGP Open.

[2] Bhatti S, Bale S, Gul S (2024). The impact of leadership style in team-based primary care - staff satisfaction and motivation. BJGP Open.

[3] Fox MN, Dickson JM, Burch P (2024). Delivering relational continuity of care in UK general practice: a scoping review. BJGP Open.

[4] Arroyave Caicedo NM, Parry E, Arslan N, Park S (2024). Integration of social determinants of health information within the primary care electronic health record: a systematic review of patient perspectives and experiences. BJGP Open.

[5] Leighton C, Cooper A, Porter A (2024). Effectiveness and safety of asynchronous telemedicine consultations in general practice: a systematic review. BJGP Open.

[6] Norori N, de Biase C, Wong YH (2024). Evaluating whether Prostate Cancer UK’s risk checker is a help or hindrance to prostate-specific antigen testing policy: a mixed-methods study. BJGP Open.

[7] Clough RAJ, Sparkes WA, Clough OT (2024). Transforming healthcare documentation: harnessing the potential of AI to generate discharge summaries. BJGP Open.

[8] NHS England (2025). General Practice ST1 recruitment figures. https://medical.hee.nhs.uk/medical-training-recruitment/medical-specialty-training/general-practice-gp/how-to-apply-for-gp-specialty-training/gp-recruitment-data/general-practice-st1-recruitment-figures.

[9] Tiffin PA, Morley E, Paton LW, Patterson F (2024). New evidence on the validity of the selection methods for recruitment to general practice training: a cohort study. BJGP Open.

[10] Homan K, Seeley R, Fisher L (2024). Safety of direct-acting oral anticoagulant (DOAC) prescribing: OpenSAFELY-TPP analysis of 20.5 million adults’ electronic health records. BJGP Open.

[11] Sætre H, Skow M, Vik I (2024). Acute cystitis in men – a nationwide study from primary care: antibiotic prescriptions, risk factors, and complications. BJGP Open.

